# High-Fidelity rPPG Waveform Reconstruction from Palm Videos Using GANs

**DOI:** 10.3390/s26020563

**Published:** 2026-01-14

**Authors:** Tao Li, Yuliang Liu

**Affiliations:** 1 Optoelectronic System Laboratory, Institute of Semiconductors, Chinese Academy of Sciences, Beijing 100083, China; litao26@semi.ac.cn; 2 Center of Materials Science and Optoelectronics Engineering, University of Chinese Academy of Sciences, Beijing 100049, China

**Keywords:** remote photoplethysmography (rPPG), waveform reconstruction, generative adversarial network (GAN), physiological signal monitoring, peak-aware loss

## Abstract

Remote photoplethysmography (rPPG) enables non-contact acquisition of human physiological parameters using ordinary cameras, and has been widely applied in medical monitoring, human–computer interaction, and health management. However, most existing studies focus on estimating specific physiological metrics, such as heart rate and heart rate variability, while paying insufficient attention to reconstructing the underlying rPPG waveform. In addition, publicly available datasets typically record facial videos accompanied by fingertip PPG signals as reference labels. Since fingertip PPG waveforms differ substantially from the true photoplethysmography (PPG) signals obtained from the face, deep learning models trained on such datasets often struggle to recover high-quality rPPG waveforms. To address this issue, we collected a new dataset consisting of palm-region videos paired with wrist-based PPG signals as reference labels, and experimentally validated its effectiveness for training neural network models aimed at rPPG waveform reconstruction. Furthermore, we propose a generative adversarial network (GAN)-based pulse-wave synthesis framework that produces high-quality rPPG waveforms by denoising the mean green-channel signal. By incorporating time-domain peak-aware loss, frequency-domain loss, and adversarial loss, our method achieves promising performance, with an RMSE (Root Mean Square Error) of 0.102, an MAPE (Mean Absolute Percentage Error) of 0.028, a Pearson correlation of 0.987, and a cosine similarity of 0.989. These results demonstrate the capability of the proposed approach to reconstruct high-fidelity rPPG waveforms with improved morphological accuracy compared to noisy raw rPPG signals, rather than directly validating health monitoring performance. This study presents a high-quality rPPG waveform reconstruction approach from both data and model perspectives, providing a reliable foundation for subsequent physiological signal analysis, waveform-based studies, and potential health-related applications.

## 1. Introduction

Photoplethysmography (PPG) is a physiological sensing technology that detects pulsatile blood volume changes based on optical interactions between incident light and subcutaneous blood flow. It is widely used for monitoring key vital signs such as heart rate, heart rate variability, and blood oxygen saturation. Traditional PPG systems rely on LED light sources and photodetectors, which must maintain direct contact with the skin to record light-intensity variations modulated by pulsatile blood flow. However, in settings such as burn care, intensive care [1], neonatal monitoring, and long-term continuous health management, the use of contact-based sensors may cause skin irritation, discomfort, or interference with clinical procedures, thereby limiting their applicability.

To overcome these limitations, video-based remote photoplethysmography (rPPG) has attracted significant attention in recent years. rPPG captures subtle optical fluctuations in skin color induced by cardiac cycles using a conventional camera, enabling non-contact, low-cost, and easily deployable physiological parameter monitoring.

However, current rPPG research still faces several limitations. First, most existing methods focus on estimating specific physiological metrics such as heart rate and heart rate variability, while largely overlooking the reconstruction of the full rPPG waveform. Second, publicly available datasets typically use fingertip pulse oximeter readings as ground-truth reference signals for facial videos. Due to inherent physiological differences between the fingertip and the face, their PPG waveforms differ substantially in multiple aspects, including the steepness of the systolic upstroke, the position and depth of the dicrotic notch, the amplitude distribution of the pulse wave, and the characteristics of pulse-wave propagation [2]. Such label mismatches caused by anatomical inconsistencies can constrain the upper-bound performance of deep learning models, making it difficult for them to learn physiologically meaningful periodic waveform features. Although this issue has profound implications for waveform-level rPPG reconstruction, it has not yet received sufficient attention in the existing literature.

To address the above issues, we developed a dual-channel PPG acquisition system capable of synchronously recording PPG signals from two different anatomical locations. Experimental validation using this system revealed that the PPG waveform morphology obtained from the radial artery at the wrist closely matches that captured from the palm, making it suitable for training neural network models. Based on this finding, we constructed a paired dataset consisting of hand-region video and wrist-based contact PPG signals, achieving high physiological consistency between the video data and the reference labels. Furthermore, we propose a generative adversarial network (GAN)-based pulse-wave synthesis framework that produces high-quality rPPG waveforms by denoising the mean green-channel signal. By integrating time-domain peak-aware loss, frequency-domain loss, and adversarial loss, our method achieves strong performance, with a root mean square error (RMSE) of 0.102, a mean absolute percentage error (MAPE) of 0.028, a Pearson correlation of 0.987, and a cosine similarity of 0.989. These results demonstrate the capability of the proposed approach to reconstruct high-fidelity rPPG waveforms.

## 2. Related Works

In 2008, Verkruysse et al. [3] demonstrated that rPPG signals could be obtained using a consumer-grade camera. In their approach, the forehead region was manually selected as a fixed region of interest (ROI), and the pixel values within the ROI were averaged to produce time series for the RGB channels. Their analysis showed that the green-channel signal exhibited clear periodicity consistent with the pulse waveform, making it suitable for physiological parameter estimation. This pioneering work provided substantial inspiration for subsequent research in the field. Since then, various camera-based rPPG extraction methods have been proposed, which can generally be categorized into two groups: traditional signal-processing methods and deep learning-based approaches.

Among traditional approaches, two representative categories are blind source separation (BSS)-based methods and reflection-model-based methods. BSS methods treat the recorded signals as a linear mixture of the blood volume pulse (BVP) component and various noise sources. This line of work was first introduced by Poh et al. [4], who modeled the RGB channel signals as a linear combination of BVP and noise. Lewandowska et al. [5] later proposed using principal component analysis (PCA) to extract the PPG signal from the RGB channels, demonstrating performance comparable to Poh’s method. Guo et al. [6] further divided the facial ROI into multiple small subregions and applied independent vector analysis (IVA) jointly across these regions; their results showed that IVA outperformed independent component analysis (ICA). Subsequently, Qi et al. [7] incorporated spatial correlations among facial subregions into the IVA framework, achieving additional performance improvements.

Another widely used category of traditional methods is based on reflection models. The core idea of these approaches is to project the RGB signals into a specific subspace to suppress noise caused by illumination variations and motion. De Haan and Jeanne [8] first introduced a chrominance-based method that models the RGB signals, comprising three components: specular reflection, diffuse reflection, and noise. By linearly combining the chrominance signals, their method effectively projects the data onto a subspace orthogonal to the specular reflection component, thereby removing unwanted signal variations. Wang et al. [9] later proposed the Plane-Orthogonal-to-Skin (POS) method, which defines a plane orthogonal to the skin-tone vector to extract the BVP signal. Although these traditional methods have achieved considerable success in heart rate estimation, they rely on idealized assumptions and often degrade in performance under complex real-world conditions [1]. In recent years, with the rapid development of deep learning, data-driven approaches have also been applied to remote physiological parameter extraction. ŠPetlík et al. [10] designed a two-dimensional convolutional network consisting of two components: a feature extractor for rPPG signal extraction from video frames, and a heart rate estimator for predicting heart rate from the extracted rPPG signals. Chen et al. [11] proposed a convolutional network incorporating an attention mechanism, which uses normalized frame differences as input to reduce illumination and motion noise, while enabling the network to focus on regions with strong BVP signals. Yu et al. [12] introduced a three-dimensional convolutional approach for rPPG signal reconstruction, which considers temporal contextual features and achieves accurate heart rate estimation for individual subjects. In [13], the researchers employed a spatiotemporal video enhancement network to strengthen the remote photoplethysmography (rPPG) signal intensity in highly compressed videos.

The aforementioned methods primarily focus on estimating average heart rate and largely overlook the reconstruction of high-quality rPPG waveforms. In this regard, Song et al. [14] proposed a generative adversarial network (GAN)-based approach for high-quality rPPG signal reconstruction. Their method first generates preliminary rPPG signals using the CHROM algorithm, and then employs a GAN framework combined with time-domain and frequency-domain joint loss functions to produce high-fidelity rPPG waveforms. Frédéric et al. [15] reconstructed realistic rPPG waveforms by leveraging the wavelet-based time–frequency representation of coarsely extracted rPPG signals in combination with image-based deep learning methods. This work highlights the growing recognition among researchers of the importance of generating high-quality rPPG waveforms for subsequent physiological parameter analysis.

## 3. Materials and Methods

### 3.1. Data Acquisition System

In this study, we developed a dual-module hardware system for the synchronous acquisition of contact-based PPG signals and video data. The system comprises (1) a dual-channel contact PPG acquisition module, and (2) an rPPG acquisition module based on a camera and active illumination. This setup enables high-precision, synchronous recording of PPG signals from different anatomical sites, as well as the simultaneous acquisition of video and PPG signals, providing a reliable dataset for subsequent waveform reconstruction studies.

#### 3.1.1. Dual-Channel PPG Acquisition System

**Figure 1:**
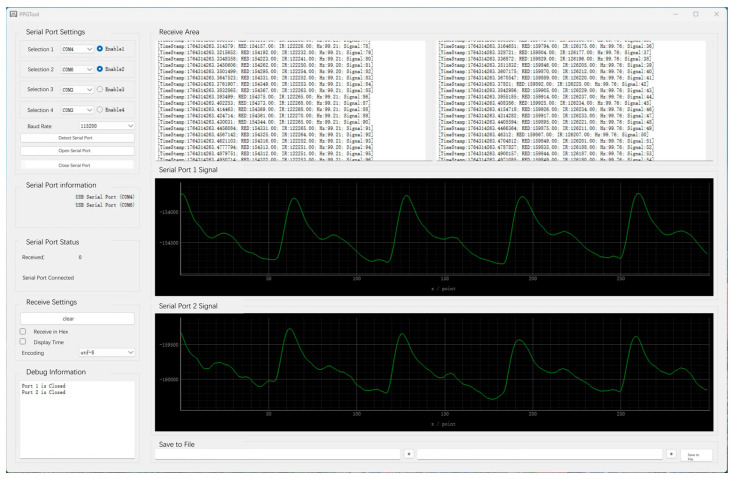
Graphical user interface (GUI) of the dual-channel PPG acquisition system.

The system employs the MAX30102 module from Maxim Integrated (Maxim Integrated Products, San Jose, CA, USA) as the photoplethysmography (PPG) sensor. In this study, the red-light channel of the MAX30102 with a wavelength of 660 nm is specifically selected for PPG signal acquisition [16], as this wavelength is widely used in contact PPG measurements and provides robust sensitivity to pulsatile blood volume changes. This sensor integrates a built-in LED light source, a photodetector, optical components, and low-noise electronics, enabling effective suppression of ambient light interference and providing high signal-to-noise ratio PPG signals. Owing to the fully integrated design of the MAX30102, the acquired data can be reliably and continuously transmitted to the microcontroller unit in real time. To achieve dual-channel synchronous sampling, this study uses the Atmel mega328p microcontroller (Atmel, San Jose, CA, USA) as the signal acquisition and processing unit. The temporal delay between the two channels is approximately 10 µs, rendering the phase difference between channels negligible. The contact PPG sensors are secured to the participants’ wrists using wristbands to ensure stable contact between the skin and the sensors, thereby minimizing motion artifacts. All acquisition units are connected to a host computer via a multi-port RS-232 hub, enabling stable serial data transmission. The host software, implemented in Python 3.9 with PySide2, supports real-time display, waveform monitoring, and data storage for up to two signal channels. The software user interface is shown in Figure 1. This acquisition system allows simultaneous recording of PPG waveforms from two different anatomical sites, providing a hardware foundation for validating waveform differences across physiological locations.

#### 3.1.2. Video Signal Acquisition System

The video acquisition system primarily consists of a camera, a green-light illumination source, and a polarizer setup. The camera used is the MV-CS016-10UC, featuring 1.6 megapixels and a maximum frame rate of 165 fps, which satisfies the high temporal resolution requirements for rPPG extraction. The illumination source is an LED array (532 nm, 4 W), which is a wavelength that exhibits a high AC/DC ratio in skin blood flow-modulated signals, facilitating the acquisition of more stable rPPG signals. To suppress specular reflections from the skin surface while preserving backscattered light modulated by blood flow, a pair of orthogonally oriented polarizers is installed in front of both the light source and the camera lens, significantly reducing motion artifact interference.

### 3.2. Data Acquisition

A total of 12 healthy adult volunteers (age: 24 ± 2 years) were recruited for this study. All participants had no known cardiovascular conditions or skin injuries that could affect signal quality. The experimental procedures and potential risks were thoroughly explained to the volunteers prior to data collection, and written informed consent was obtained from all participants. This study was approved by the Ethics Committee of the University of Chinese Academy of Sciences (Approval No. UCASSTEC25-046), and all experiments were conducted in accordance with the Declaration of Helsinki. The study was conducted solely for scientific research purposes and involved no commercial conflicts of interest.

#### 3.2.1. Synchronous Acquisition of Wrist and Palm PPG Signals

**Figure 2:**
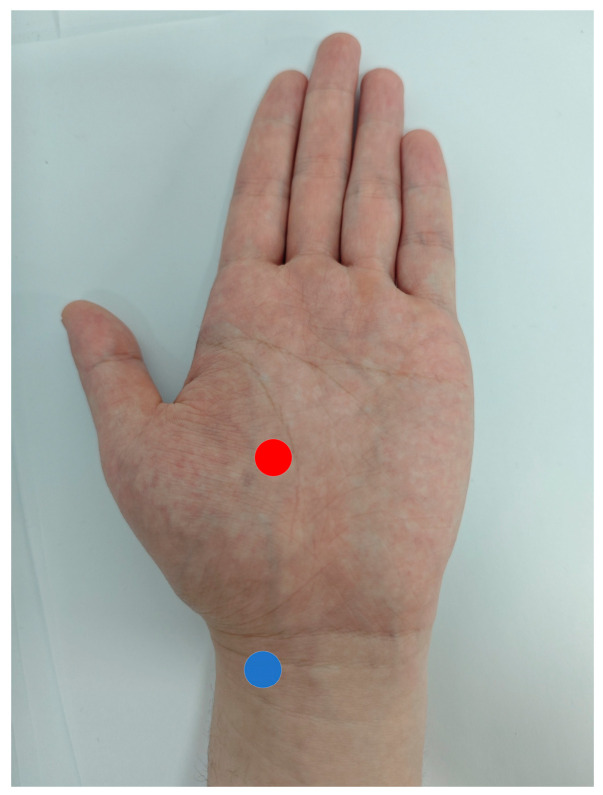
The figure illustrates the recording sites for dual-channel PPG acquisition, with red dots indicating the palm collection locations and blue dots indicating the wrist collection locations.

To evaluate the feasibility of using wrist PPG signals as reference labels for palm-region videos, PPG signals were recorded according to the electrode positions illustrated in Figure 2. Each participant was instructed to rest for at least three minutes prior to the experiment to ensure stable heart rate. After sufficient rest, one-minute, dual-channel PPG recordings were acquired from each participant using the aforementioned sensor system, with a sampling rate of 100 Hz. The purpose of this data acquisition was to verify whether the collected PPG reference signals are physiologically consistent with the PPG signals in the regions used for rPPG extraction, thereby confirming their suitability for training neural network models.

#### 3.2.2. Synchronous Acquisition of Video Data and PPG Reference Signals

To collect a dataset for neural network training and validation, PPG sensors were secured to the participants’ wrists using wristbands while a camera recorded the palm region. During data acquisition, the camera was positioned approximately 40 cm away from the palm and oriented perpendicular to the palm plane. All recordings were conducted in a darkroom environment without ambient light interference. A 532 nm light source was used to illuminate the palm, arranged in parallel with the camera’s optical axis to ensure uniform illumination conditions. Each participant was instructed to rest for at least three minutes prior to the experiment to ensure a stable heart rate. Following rest, 20 s recordings were acquired from each participant using the aforementioned hardware system, with both the camera and PPG sensors operating at a sampling rate of 100 Hz. Each participant completed 20 recording sessions, resulting in a total of 240 data segments. The dataset was subsequently split into training and validation sets at a ratio of 9:1, serving to train the neural network models and evaluate their performance. It should be noted that during data acquisition, participants maintained a natural and relaxed posture without being explicitly instructed to remain completely still. As a result, the video data inevitably contained a certain degree of motion artifacts, primarily manifested as slight shifts in the palm position, local non-rigid deformations caused by natural muscle relaxation or tension, and low-amplitude periodic movements induced by respiration. Overall, the magnitude of these motions was small and did not involve large-scale hand displacements or rapid changes in posture.

### 3.3. rPPG Waveform Reconstruction Framework

**Figure 3:**
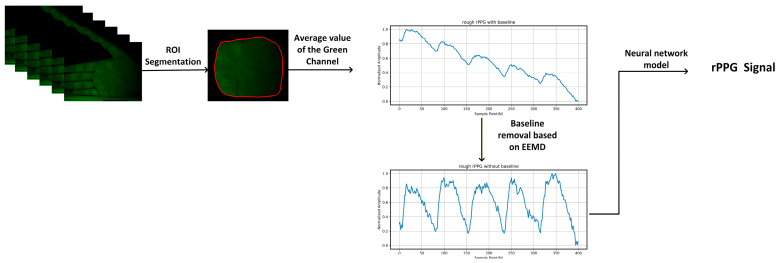
The rPPG waveform reconstruction framework.

The overall algorithmic workflow is illustrated in Figure 3. Our method first employs a lightweight semantic segmentation network, as proposed by Yang et al. [17], to extract the region of interest (ROI). The mean of the green-channel pixels within the extracted ROI is then computed to convert a video sequence containing only the palm region into a one-dimensional temporal signal, representing a preliminary, coarse rPPG signal. The specific extraction method is expressed as follows (Equation (1)):

(1) $${rPPG}_{raw}\left(t\right)=\frac{\sum_{i=1}^{i=N}I_{i}(t)}{N}$$

where ${rPPG}_{raw}\left(t\right)$ denotes the mean value of the green-channel pixels within the ROI at time t, $I_{i}(t)$ represents the green-channel intensity of the i-th pixel within the ROI at time t, and N is the total number of pixels in the ROI.

To suppress low-frequency baseline drift and slow-varying trends introduced by illumination fluctuations and physiological motion, the rough rPPG signal is further processed using an ensemble empirical mode decomposition (EEMD)-based baseline removal algorithm. In this procedure, the signal is decomposed into intrinsic mode functions (IMFs) using 50 ensemble trials with a Gaussian noise width of 0.01, which represents a compromise between decomposition stability and computational efficiency. The maximum number of IMFs is limited to eight to avoid over-decomposition while retaining sufficient temporal detail. Low-frequency IMFs corresponding to baseline drift are identified based on their peak density, with components whose number of local extrema is lower than 0.7 times the expected cardiac cycle count (signal length divided by sampling frequency) being removed. The remaining IMFs are summed to reconstruct a baseline-corrected rPPG signal, and the mean value difference between the reconstructed and original signals is compensated to preserve the overall signal amplitude. The effect of baseline removal, showing the comparison between the original and corrected signals, can be seen in Figure 3. This initial signal is subsequently fed into the proposed neural network model for waveform reconstruction.

The neural network model used in this study is based on a generative adversarial network (GAN) architecture. GANs are deep learning models trained using a game-theoretic approach, in which a generator and a discriminator are jointly optimized through an adversarial process [18]. In this task, the discriminator distinguishes between real PPG signals and generated synthetic PPG signals, while the generator aims to produce realistic PPG signals from coarse rPPG inputs, making it difficult for the discriminator to differentiate between them, thereby enabling the recovery of rPPG signals to true PPG signals.

**Figure 4:**
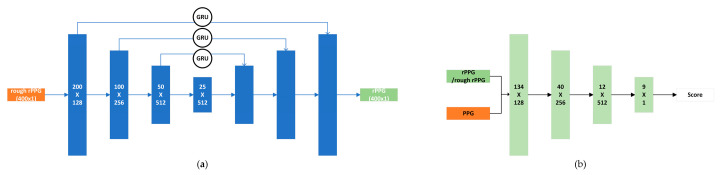
Neural network architecture: (**a**) the generator network; (**b**) the discriminator network.

As illustrated in Figure 4a, our generator adopts a U-Net architecture, originally proposed by Ronneberger et al. [19] for medical image segmentation. The U-Net consists of a contracting path (encoder) and an expansive path (decoder), forming the characteristic U-shaped structure. In this study, the generator is implemented as a one-dimensional convolutional U-Net. The input signal is first progressively compressed to a bottleneck layer through down-sampling layers and then gradually restored to its original length via up-sampling layers. Convolutional layers in both the encoder and decoder employ repeated padded convolutions with a kernel size of four and a stride of two; all layers except the final layer are activated using PReLU [20] and normalized via instance normalization (IN) [21]. The decoder utilizes transposed convolutions to progressively recover the sequence length and applies a Tanh activation function in the final layer. To fully leverage the feature information extracted by the encoder, skip connections are introduced between the contracting and expansive paths to fuse global features. Additionally, to enhance the temporal consistency of the signal during propagation, gated units [22] are incorporated into the skip connections, further improving the temporal coherence of the generated signals.

As shown in Figure 4b, the discriminator in this study is composed of several stacked one-dimensional convolutional layers, each with a kernel size of five and a stride of three, except for the final layer, which uses a stride of one and a linear activation function. All convolutional layers except the last employ LeakyReLU activation [23] and are combined with instance normalization (IN) to accelerate network convergence. The discriminator takes a two-channel input, where the rough rPPG signal serves as a conditional input to guide the discriminator in evaluating the generated signal. Specifically, the discriminator distinguishes between pairs of generated waveforms (generated PPG, rough rPPG) and corresponding reference waveform pairs (PPG, rough rPPG). Its output represents the probability of the input being classified as real, thereby providing optimization feedback to the generator during adversarial training.

For the design of the loss function, our goal is to generate the rPPG signal from the preliminarily extracted rough rPPG input signal, such that rPPG closely matches the reference signal. Achieving this requires training our neural network model on a large set of paired data. Given that PPG signals exhibit prominent characteristics in both the time and frequency domains, drawing inspiration from the work of Chen et al. [14] and, in conjunction with our proposed framework, define the following loss functions separately in the time and frequency domains to enable the network to better capture the features of PPG signals:

(2) $$L_{G}=\frac{1}{2}{\left(D\left(rPPG,{rPPG}_{rough}\right)-1\right)}^{2}+\alpha {\left\|PPG-rPPG\right\|}_{1}+\beta {\left\|{PPG}_{f}-{rPPG}_{f}\right\|}_{1}+\gamma \left\|{PPG}_{peaks}-{rPPG}_{peaks}\right\|$$

(3) $$L_{D}=\frac{1}{2}{\left(D\left(rPPG,{rPPG}_{rough}\right)\right)}^{2}+\frac{1}{2}{\left(D\left(PPG,{rPPG}_{rough}\right)-1\right)}^{2}$$

**Figure 5:**
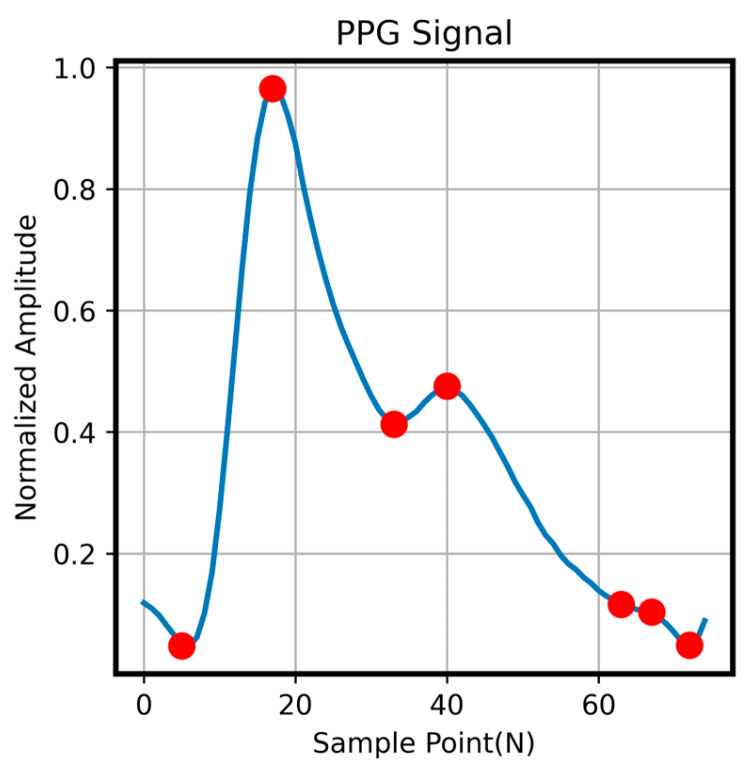
Schematic of characteristic peak points in a PPG signal.

The generator loss $L_{G}$ consists of three components: the first is an adversarial loss similar to that used in the least squares GAN (LSGAN) [24]; the second is a time-domain waveform reconstruction loss; and the third is a frequency-domain spectral loss, which further refines the previous two components. As shown in Figure 5, since the characteristic inflection points of PPG waveforms reflect cardiovascular activity, we annotate the key peaks in the PPG signal and compute a separate loss at these points to enhance the accuracy of the generated signal at physiologically important locations. The discriminator loss follows the LSGAN formulation to enforce the distinction between generated and reference signals. For the spectral loss, both rPPG and reference PPG signals are transformed using a 1024-point fast Fourier transform (FFT) to obtain their corresponding spectral representations ${PPG}_{f}$, ${rPPG}_{f}$, where ${\left\|.\right\|}_{1}$ denotes the L1 norm. The hyperparameters α and *β* in the generator loss correspond to the weights of the time-domain waveform loss and frequency-domain spectral loss, respectively, while *γ* represents the weight for the characteristic peak loss. In this study, the hyperparameters were set to α = 1.0, β = 0.3, and γ = 0.5. By minimizing this composite loss, the generator learns both the temporal and spectral characteristics of the signal, thereby improving the quality of the generated waveform. The GAN model is trained by alternating updates between the discriminator *D* and generator *G*, with the training epoch of *D* setting to three times that of *G*. The model is optimized using the Adam optimizer [25], with network weights initialized from a Gaussian distribution with zero mean and a standard deviation of 0.02. The entire framework is implemented in PyTorch (torch 1.11.0) and trained on an NVIDIA RTX 3090 GPU (NVIDIA, Santa Clara, CA, USA) for 1000 epochs with a batch size of 192.

## 4. Results

### 4.1. Results of Dataset Feasibility Validation

**Figure 6:**
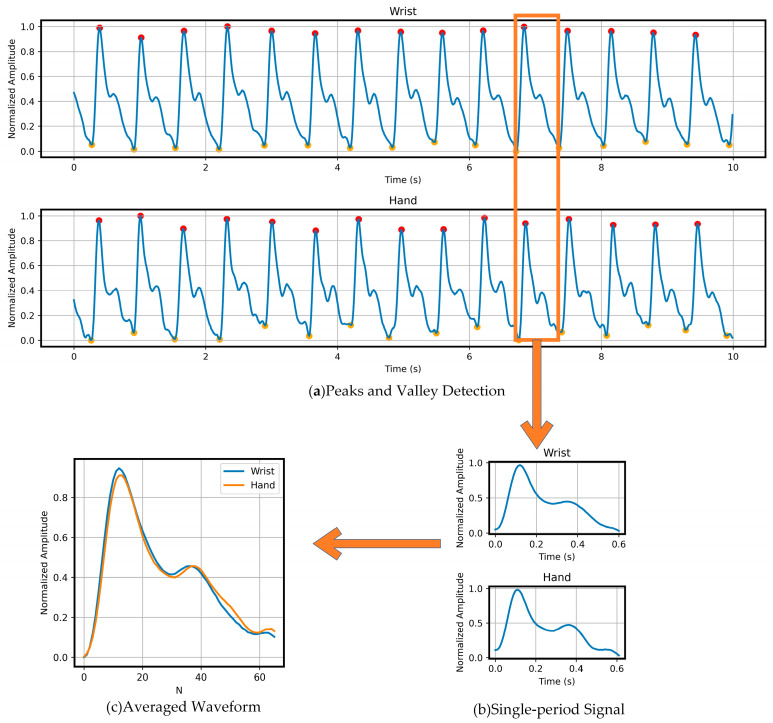
The pipeline of the PPG signal processing; (**a**) peaks and valley detection; (**b**) single-period signal; (**c**) averaged waveform.

To verify that our dataset does not exhibit the physiological discrepancies observed in previous datasets, we first used the constructed multi-channel PPG acquisition system to synchronously record PPG waveforms at the wrist and palm. Figure 6a presents a comparison of reference waveforms from a typical participant at these two measurement sites. It can be observed that the signals from both sites are largely consistent in terms of peak amplitude, the steepness of the rising edge, and the dicrotic notch structure.

To further quantify the similarity between the two sites, we followed the approach of Hartmann et al. [26] and processed the signals as follows: (1) detection of valley positions, as shown in Figure 6a; (2) segmentation of the acquired PPG signals into individual cardiac cycles based on the detected valleys, as illustrated in Figure 6b; (3) normalization of the amplitude of each single-period signal. Considering that some periods were affected by motion artifacts, all signals were manually screened to ensure quality, (4) averaging all normalized single-period signals to obtain a representative normalized waveform, as shown in Figure 6c. This waveform integrates the PPG signals from all 12 participants, providing a generalizable representation.

**Figure 7:**
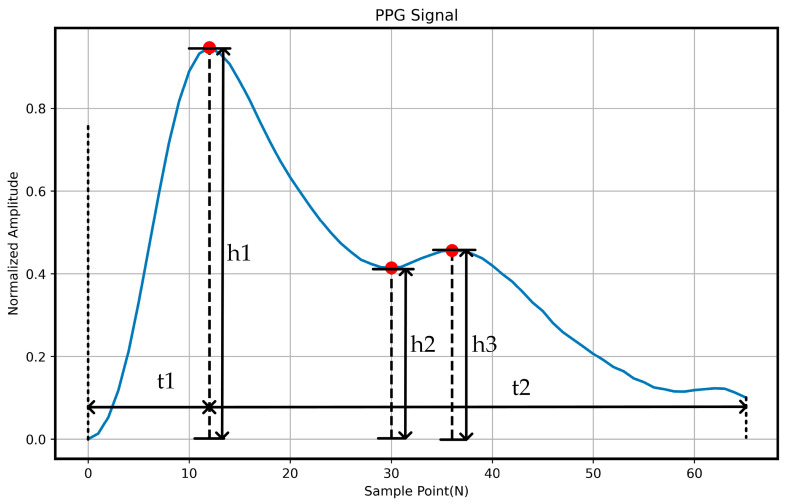
The characteristics derived from PPG waveform.

After completing the above procedures, as shown in Figure 7, we calculated several morphological indices of the PPG signals from the two measurement sites, including rise time $t_{1}$, fall time $t_{2}$, main peak amplitude $h_{1}$, dicrotic notch amplitude $h_{2}$, and second peak amplitude $h_{3}$. The overall waveform similarity was assessed by evaluating the differences in these indices as well as the Pearson correlation coefficient (ρ).

Table 1 presents the statistical comparison of PPG signals acquired from the wrist and palm measurement sites. The results indicate that the differences between the two sites in all morphological indices—including rise time $t_{1}$, fall time $t_{2}$, main peak amplitude $h_{1}$, dicrotic notch amplitude $h_{2}$, and second peak amplitude $h_{3}$—are minimal. Moreover, the Pearson correlation coefficient (ρ) between the signals from the two sites reaches 0.998, demonstrating a very high degree of waveform consistency. Compared with our previous experimental findings, this dataset exhibits an inherent physiological advantage, further confirming the reliability and feasibility of the dataset constructed in this study.

### 4.2. rPPG Signal Waveform Reconstruction Results

**Figure 8:**
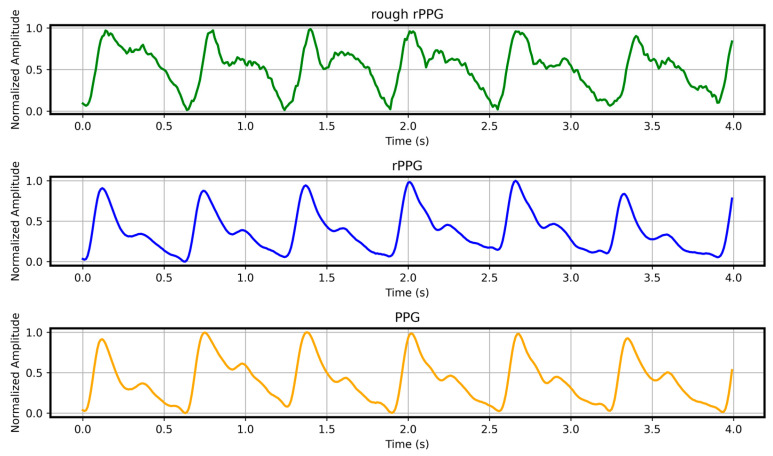
The rough rPPG, the reconstructed rPPG signals, and the corresponding reference PPG signals.

Following the proposed rPPG signal reconstruction framework, the video data were processed to obtain the reconstructed rPPG signals. A comparison between the rough rPPG, reconstructed rPPG signals, and the reference PPG signals is presented in Figure 8.

From the visual comparison, the reconstructed rPPG signal exhibits a high degree of consistency with the reference PPG signal in key morphological structures, including peak amplitude, the steepness of the rising edge, and the dicrotic notch. To quantitatively assess the reconstruction quality, we employed four evaluation metrics—mean absolute percentage error (MAPE), root mean square error (RMSE), Pearson correlation coefficient (ρ), and cosine similarity—to comprehensively analyze the similarity between the reconstructed rPPG signals and the reference PPG signals.

Table 2 presents the quantitative evaluation metrics between the reconstructed rPPG signals and the reference PPG signals. The results show that the mean absolute percentage error (MAPE) is 0.028, indicating only a small deviation in amplitude between the reconstructed and reference signals. The root mean square error (RMSE) is 0.102, further demonstrating the minimal overall waveform discrepancy. In terms of correlation measures, the Pearson correlation coefficient reaches 0.987, suggesting a high degree of linear consistency in the temporal structure of the two signals. The cosine similarity of 0.979 indicates strong alignment in the overall waveform direction.

To further validate the effectiveness of the proposed loss function applied at the temporal characteristic peaks and the gated skip-connection mechanism, we conducted a series of ablation studies. The experimental results are summarized in Table 3.

The experimental results indicate that when neither the temporal peak-specific loss nor the gated skip-connection mechanism was employed, the reconstructed signal exhibited relatively poor performance (MAPE 0.038, RMSE 0.137, Pearson 0.913, cosine similarity 0.932). Enabling only the gated skip-connection mechanism improved the overall waveform trend and temporal continuity (MAPE 0.033, RMSE 0.122, Pearson 0.941, cosine similarity 0.957), while using only the temporal peak-specific loss further enhanced the recovery of local morphological features (MAPE 0.031, RMSE 0.118, Pearson 0.964, cosine similarity 0.968). When both mechanisms were combined, the model achieved the best performance (MAPE 0.028, RMSE 0.102, Pearson 0.987, cosine similarity 0.979), with the global waveform distribution and key local features closely matching the reference, demonstrating the complementary roles of the two components in waveform-level rPPG reconstruction.

Furthermore, when adversarial training was removed (without adversarial training), although the error metrics remained better than those of models using only the peak-specific loss or the gated skip-connection mechanism (MAPE 0.030, RMSE 0.113, Pearson 0.971, cosine similarity 0.964), the overall waveform distribution and local structure consistency were noticeably degraded compared to the complete model. This indicates that adversarial training plays a critical role in capturing the global waveform morphology and enhancing the consistency of key local features, thereby validating the effectiveness of the GAN framework in high-fidelity rPPG waveform reconstruction.

Taken together, these results demonstrate that both the temporal peak-specific loss function and the gated skip-connection mechanism play significant roles in enhancing rPPG signal reconstruction accuracy. Moreover, their combined application substantially improves amplitude fidelity, waveform morphology preservation, and temporal correlation of the generated signals, thereby validating the effectiveness of the proposed method in capturing critical temporal features and global waveform information.

## 5. Discussion

Existing deep learning-based rPPG studies have primarily relied on datasets pairing facial videos with fingertip PPG signals, focusing largely on estimating physiological metrics such as heart rate, while paying limited attention to waveform reconstruction. However, physiological discrepancies between facial and fingertip PPG signals often hinder accurate waveform recovery. To address this, we constructed a paired dataset of palm-region videos and wrist PPG signals, and verified its high physiological consistency through both visual inspection and statistical analysis of key waveform metrics (Table 1). This dataset provides a reliable foundation for learning authentic rPPG waveform features and enables high-fidelity reconstruction.

It should be noted that this study does not attempt to explain the underlying mechanisms by which palm videos lead to improved rPPG waveform reconstruction performance. The observed improvements may arise from multiple factors, including the closer physiological consistency between palm videos and wrist-based contact PPG signals, the potentially higher optical signal-to-noise ratio of the palm region, and the relatively stable and convenient data acquisition setup used in this study. A detailed investigation of these factors is beyond the scope of the present work.

From a signal perspective, PPG waveforms are quasi-periodic and exhibit pronounced non-sinusoidal characteristics, with critical physiological information concentrated at specific morphological points, such as the steep systolic upstroke, the main peak, and the dicrotic notch. Conventional MSE-based training often diminishes these local features due to the dominance of smooth waveform segments. The proposed temporal peak-specific loss assigns higher weights to these key regions, guiding the model to preserve physiologically meaningful details. Ablation studies indicate that removing this loss reduces waveform reconstruction quality, confirming its independent contribution to capturing waveform morphology.

From a network design perspective, skip connections help retain low-level temporal features but may propagate noise in rPPG signals. Introducing gated skip connections enables the network to selectively modulate feature flow across time and channels, preserving important temporal structures while suppressing irrelevant disturbances. The complementary effects of peak-aware loss and gated skip connections enhance both local morphological fidelity and global waveform consistency, as reflected in improved MAPE, RMSE, and correlation metrics in ablation experiments (Table 3).

From a modeling perspective, both the peak-aware loss and spectral loss are based on explicitly defined distance metrics, constraining the signal consistency at key temporal points and in the frequency domain, respectively. However, these losses are limited in capturing higher-order morphological and statistical characteristics inherent in PPG waveforms. In contrast, adversarial training introduces a discriminator to constrain the generated signals at the distribution level, encouraging the generator to learn the statistical patterns of real PPG waveforms beyond pointwise errors or spectral matching. Therefore, in this study, the GAN primarily serves as a distribution-level regularization, complementing traditional losses in modeling waveform morphology, rather than independently determining reconstruction performance. It should be noted that we do not attempt a systematic comparison between the proposed GAN framework and recent non-GAN time-series modeling methods. While such approaches are also valuable for rPPG waveform modeling, they fall outside the scope of this study, which focuses on waveform-level reconstruction with an emphasis on loss design and data consistency.

Although this study focuses on waveform reconstruction rather than direct physiological parameter extraction, the proposed framework generates high-quality rPPG signals suitable for downstream health monitoring applications. The current dataset and experiments primarily address scenarios with mild motion artifacts and do not encompass extreme movements such as substantial hand translation, rotation, rapid motion, or complex non-rigid deformations. Consequently, the robustness of the proposed method under strong motion interference remains to be systematically evaluated. Additional limitations include the exclusive recruitment of healthy young adults, the use of 532 nm video illumination in combination with 660 nm contact PPG signals, and the lack of clinical validation. Future work will expand the dataset to include more diverse populations and complex motion patterns, explore wavelength-matched acquisitions, and integrate motion compensation or advanced temporal modeling strategies. These efforts aim to further improve waveform fidelity and extend the model’s applicability in realistic, unconstrained health monitoring scenarios.

In summary, by combining a physiologically consistent dataset with a model emphasizing peak-aware loss and gated skip connections, this study establishes a robust approach for high-fidelity rPPG waveform reconstruction, providing a reference framework for waveform-level physiological signal analysis.

## 6. Conclusions

In this study, we constructed a dataset comprising palm-region videos paired with wrist-based contact PPG signals and validated the high morphological consistency between the two measurement sites through physiological consistency analysis, effectively mitigating the physiological mismatches commonly observed in previous datasets based on facial videos and fingertip PPG signals. Building upon this dataset, we propose a generative adversarial network (GAN)-based neural network model for reconstructing high-fidelity waveforms from initially extracted coarse rPPG signals. The model employs a U-Net generator and incorporates gated units within the skip connections to enhance temporal information propagation, while applying a specialized loss function at key waveform peaks to enable more precise feature capture. Experimental results demonstrate that the proposed method significantly improves waveform morphology similarity, and ablation studies further confirm the critical roles of both the gated skip-connection mechanism and the peak-specific loss function in signal reconstruction. It should be emphasized that this study focuses exclusively on waveform-level reconstruction of rPPG signals and does not aim to evaluate overall rPPG performance in facial video scenarios or to assess downstream physiological parameter estimation tasks such as heart rate or heart rate variability. Therefore, the reported results should be interpreted as evidence of reconstruction fidelity rather than a comprehensive evaluation of rPPG application performance. Nevertheless, this study presents a high-fidelity rPPG waveform reconstruction approach from both data and model perspectives, offering a reference framework for future applications of waveform-level reconstruction methods in clinical physiological signal analysis.

## Figures and Tables

**Table 1:** Results of dataset feasibility validation.

$∆t_{1}(ms)$	$∆t_{2}(ms)$	$∆h_{1}$	$∆h_{2}$	$∆h_{3}$	ρ
0.27	0.19	0.031	0.013	0.005	0.998

**Table 2:** Reconstruction results of rPPG signals.

MAPE	RMSE	ρ	Cosine Similarity
0.028	0.102	0.987	0.979

**Table 3:** Results of ablation studies.

Method	MAPE	RMSE	ρ	**Cosine Similarity**
Without peaks and gru ^1^	0.038	0.137	0.913	0.932
Only gru ^2^	0.033	0.122	0.941	0.957
Only peaks ^3^	0.031	0.118	0.964	0.968
With peaks and gru ^4^	**0.028**	**0.102**	**0.987**	**0.979**
Without adversarial training ^5^	0.030	0.113	0.971	0.964

^1^ Without incorporating either the temporal peak-specific loss function or the gated skip-connection mechanism. ^2^ With only the gated skip-connection mechanism enabled. ^3^ With only the temporal peak-specific loss function applied. ^4^ With both the temporal peak-specific loss function and the gated skip-connection mechanism fully incorporated. ^5^ Compared to the complete model, removing adversarial training.

## Data Availability

Due to restrictions imposed by the Institutional Ethics Committee, the relevant data can be accessed upon reasonable request to the corresponding author.
